# Risk Assessment of Essential and Toxic Elements in Freshwater Fish Species from Lakes near Black Sea, Bulgaria

**DOI:** 10.3390/toxics10110675

**Published:** 2022-11-08

**Authors:** Katya Peycheva, Veselina Panayotova, Rositsa Stancheva, Lubomir Makedonski, Albena Merdzhanova, Vincenzo Parrino, Vincenzo Nava, Nicola Cicero, Francesco Fazio

**Affiliations:** 1 Department of Chemistry, Medical University of Varna, 9002 Varna, Bulgaria; 2 Department of Chemical, Biological, Pharmaceutical and Environmental Sciences, University of Messina, 98100 Messina, Italy; 3 Department of Biomedical and Dental Sciences and Morphofunctional Imaging, University of Messina, 98100 Messina, Italy; 4 Science4life srl, Spin off Company, University of Messina, 98100 Messina, Italy; 5 Department of Veterinary Sciences, University of Messina, Polo Universitario Annunziata, Viale Palatucci snc, 98100 Messina, Italy

**Keywords:** heavy metals, freshwater fish, target hazard quotient, hazard index, target risk

## Abstract

The aims of this study were to measure the concentrations of selected toxic and essential elements in the muscle tissue of five common freshwater fish species (roach (Rutilus rutilus), freshwater bream (Abramis brama), prussian carp (Carassius gibelio), crucian carp (Carassius carassius) and common carp (Cyprinus carpio)) from Lake Burgas and Lake Mandra (Bulgaria). In all samples the levels of Cd, Cr, Cu, Mn, Ni, Pb, Fe and Zn were under the maximum allowed concentrations for safe human consumption in Bulgaria and ranged as follows: Cd 0.02–0.05; Cr 0.03–0.06; Cu 0.11–0.20; Mn 0.05–0.71; Ni 0.06–0.11; Pb 0.15–0.27, Fe 1.68–5.86 and Zn 1.94–9.06 mg/kg wet weight. The concentration of As was under detection limit. An assessment of the human risk by calculation of the target hazard quotients (THQ), hazard index (HI) and target risk (TR) was performed. The target hazard quotient (THQ) for individual elements and HI for combined metals were lower than 1, indicating no health risk for consumers due to the intake of either individual or combined metals. The target risk for iAs, Pb and Ni was below 10^−6^, indicating no carcinogenic risk. According to these results, the consumption of these freshwater fish species is safe for human health.

## 1. Introduction

A matter of concern over the past few decades is the contamination of freshwaters with a wide range of pollutants such as heavy metals [1,2,3,4,5,6,7,8]. Heavy metal contaminants are of particular concern due to their high toxicity, persistence, and ability to accumulate into the food chain, reaching human beings [9,10,11,12,13,14,15,16,17,18]. Metals such as iron, copper, zinc and manganese are essential since they play an important role in biological systems, whereas nickel, lead and cadmium are considered non-essential metals, as they are toxic, even in trace amounts [19,20]. Toxic effects can also be observed if the essential element’s level is excessively elevated [4,20]. It is of great importance to determine the element composition of fish organisms inhabiting the water bodies, regarding the content of heavy metal in order to evaluate the possible risks associated with their human health consumption [21].

Fish is widely consumed worldwide as it provides high-quality proteins, polyunsaturated fatty acids, vitamins and other essential nutrients [22]. For local inland residents, freshwater fish is a valuable source of protein [16]. On the contrary, fish species are constantly exposed to various contaminants via the surrounding aquatic medium. Accumulation of toxic compounds in different aquatic species and their tissue depends on several factors such as physiological condition, their habitat, fat content, capacity to adapt and biometric characteristics [1]. This is one of the reasons why fishes have been commonly used as good bioindicators of heavy metal pollution as they may accumulate metals by direct absorption from aquatic systems [23,24].

Lake Burgas (Vaya Lake) and Lake Mandra are located in the immediate proximity of the Black Sea, Bulgaria. Lake Burgas is the largest natural lake in Bulgaria. It is connected to the Black Sea via narrow channels, and due to the inflow of three rivers its brackish waters are characterized by low salinity with significant annual fluctuations. Lake Mandra was a brackish natural lake until 1963, when it was turned into a reservoir with the construction of a dam to secure fresh water for a large oil refinery. They are economically important for the southeastern region of the country since they are used for recreational, irrigating and fish producing purposes. The waters from the two lakes flow into the Black Sea basin. Rapid industrial and economic development has occurred around the lakes over the past few decades, leading to anthropogenic contamination of the aquatic environment. Several studies have confirmed the water quality of the lakes and some pollutants, [25,26,27] but to our knowledge studies on the bioaccumulation of metals in fishes from Lake Burgas and Mandra lake are scarce. 

The specific objectives of this study were: (1) to determine the levels of some toxic (As, Cd, Ni and Pb) and essential (Cr, Cu, Fe, Mn and Zn) elements in muscle tissues of five freshwater fish species (roach (*R. rutilus*), freshwater bream (*A. brama*), prussian carp (*C. gibelio*), crucian carp (*C. carassius*) and common carp (*C. carpio*)) collected from Lake Bourgas and Lake Mandra (Bulgaria) and (2) to assess the potential human health risk associated from freshwater fish consumption in two target groups (males and females) by calculating the target hazard quotients (THQ), hazard index (HI) and target risk (TR).

## 2. Materials and Methods

### 2.1. Sampling and Sample Treatment 

Samples of freshwater fishes were randomly acquired by local fishermen by nets from Lake Mandra and Lake Bourgas across the coastal waters of Bulgarian Black Sea. All the fish species were randomly sampled from February to November 2021. These sampling sites are 42° 25′ 14.39″ N (Station 1) and 42° 29′ 59.99″ N (Station 2).

Samples were immediately transferred to the laboratory in foam boxes filled with ice. The five species (19 samples) included in this study are shown in Table 1. Total length and weight of the samples were measured to the nearest millimeter and gram before dissection. For sample preparation, only filets of the edible part of each individual were used. Approximately, 1.0 g samples of muscle from each fish were dissected, washed with distilled water, weighted, packed in polyethylene bags and stored at least –18 °C until chemical analysis.

### 2.2. Reagents and Standard Solutions

All solutions were prepared with analytical reagent grade chemicals and ultra-pure water (18 MΩ cm) generated by purified distilled water with a Millipore Milli-Q Gradient A-10 water purification system (Bedford, MA, USA). All the plastic and glassware were cleaned by soaking in 2 M HNO_3_ for 48 h, and rinsed five times with distilled water, and then five times with deionized water prior to use. The calibration standard solutions were freshly prepared by dilution of Optima Family Multi-Element Standard, Matrix per Volume: 2% HNO_3_ and Multi-Element Calibration Standard 3, Matrix per Volume: 5% HNO_3_ stock solution (PerkinElmer^®^, Waltham, MA, USA). A DORM-2 (NRCC, Ottawa, ON, Canada) certified dogfish tissue was used as the calibration verification standard. Recoveries between 90.5 and 108% were accepted to validate the calibration.

### 2.3. Sample Digestion and Instrumental Parameters

Fish samples were thoroughly washed with Milli-Q water. The fish specimens were dissected using a Teflon knife and samples of fish filets quickly removed and washed again with Milli-Q water. Wet digestions were performed in triplicate by weighing approximately 1.0 g of the fish tissues with a mixture of 10 mL HNO_3_ (65% Merck, Suprapur, Darmstadt, Germany) in a microwave digestion system MARS 6 (CEM Corporation, Matthews, NC, USA) (Table 2). The digested samples were diluted to 25 mL with Milli-Q water and stored in polyethylene bottles. A blank digest was performed in the same way. The concentrations of As, Cd, Cr, Cu, Fe, Mn, Ni, Pb, Zn in the samples were determined using ICP-OES Spectrometer (Optima 8000, Perkin Elmer, Waltham, MA, USA) (Table 3).

### 2.4. The Public Health Risk Evaluation Associated with Freshwater Fish Consumption

#### 2.4.1. Target Hazard Quotient (*THQ*)

The *THQ* is used to determine the non-carcinogenic risk level due to pollutant exposure. To assess the health risk from metal contaminated fishes, the *THQ* was calculated as per USEPA Region III Risk Based Concentration Table [28] by using the following equation:

(1) $$THQ=\frac{\left(M_{C}\times I_{R}\times 10^{-3}\times \;EF\times ED\right)}{RfD\times BW_{a}\times AT_{n}}$$

where *M_C_* is toxic or essential element concentration in muscle tissue of fish species (mg/kg ww), *I_R_* is the fish ingestion rate (13.7 g/kg dw) [29], *EF* is the exposure frequency or number of exposure events per year of exposure (365 days/year), *ED* is the exposure duration (30 years or 10 950 days) for non-cancer risk as used by USEPA [28], *RfD* is the reference dose of individual metal (0.3 μg/kg bw/day for As, 1 μg/kg bw/day for Cd, 40 μg/kg bw/day for Cu, 3 μg/g day for Cr, 9 μg/g day for Fe, 20 μg/kg bw/day for Ni, 3.5 μg/kg bw/day for Pb, 140 μg/kg bw/day for Mn and 300 μg/kg bw/day Zn), *BW_a_* is an average adult body weight (two target groups: 65 kg for females and 79 kg for males) and *AT_n_* is the average exposure time for non-carcinogens (10,950 days) [28].

*THQ* is an integrated risk index that compares the ingested amount of a contaminant with a standard reference dose [30]. The assessment of health risk is carried out based on assumptions. The acceptable value for *THQ* equals or is less than 1 according to USEPA [28].

#### 2.4.2. Hazard Index (*HI*)

The hazard index from THQs is expressed as the total of the hazard quotients [28]:

(2) $$HI=THQ_{As}+THQ_{Cd}+THQ_{Cu}+THQ_{Cr}+THQ_{Fe}+THQ_{Ni}+THQ_{Pb}+THQ_{Mn}+THQ_{Zn}$$

#### 2.4.3. Target Risk (*TR*)

Target cancer risk (*TR*) indicates carcinogenic risks. The model for estimating *TR* was shown as follows:

(3) $$TR=\frac{M_{c}\times I_{R}\times 10^{-3}\times CPS_{o}\times EF\times ED}{BW_{a}\times AT_{c}}$$

where *M_C_* is the toxic or essential element concentration in muscle tissue of fish species (mg/kg ww), *I_R_* is the fish ingestion rate (13.7 g/kg dw) [29]; *CPS_o_* is the carcinogenic potency slope, oral (As = 1.5 mg/kg bw-day, Ni = 1.7 mg/kg bw-day, Pb = 0.0085 mg/kg bw-day); *EF* is the exposure frequency (365 days/year), *ED* is the exposure duration (30 years or 10,950 days), *BW_a_* is an average adult body weight (two target groups: 65 kg for females and 79 kg for males), *AT_c_* is the averaging time, carcinogens (day/years) (25,550) and was calculated by multiplying exposure frequency in exposure duration over lifetime.

*TR* value for intake of As, Ni and Pb was calculated to indicate the carcinogenic risk since Cd, Cr, Cu, Fe, Mn and Zn do not cause any carcinogenic effects. *TR* value is a term used to assess cancer risk and define the probability of an individual developing any type of cancer from lifetime exposure to carcinogenic hazards. According to the US EPA risk management guidelines, the value of acceptable risk is between 1 × 10^−6^ and 1 × 10^−4^ [31].

### 2.5. Statistical Analysis

All analyses were performed in triplicate and the results were expressed as mean ± standard deviation (SD). The results for toxic and essential elements were stated as mg/kg w.w. *t* test was used to compare the results for heavy metals composition. Differences at *p* ≤ 0.05 were considered significant (Graph Pad Prism 6).

## 3. Results and Discussion

### 3.1. Heavy Metals Concentration in Freshwater Fish Muscle Tissues

Mean concentrations and standard deviations of the analyzed heavy metals of the five fish species from the south-eastern region of Bulgaria are shown in Table 4. The summarized results of this study are expressed as means (mg/kg) wet weight (ww). The concentration of As in all samples was under limit of detection.

The primary source of cadmium exposure is from the food supply, especially contaminated fish and fish products. A higher amount of Cd in the body can lead to kidney failure and lung cancer [37]. The concentration of Cd in the analyzed fish species ranged from 0.02 mg/kg ww for *A. brama* up to 0.05 mg/kg ww for *R. rutilus*. Cd is an element capable of producing chronic toxicity present at minimal concentration of 1 mg/kg [38]. The maximum Cd level permitted for muscle meat of fish, excluding some marine species, is 0.05 mg/ kg ww, according to the European Community and Bulgarian Food Regulation [39]. Cadmium concentration concerning the freshwater fish species in literature has been reported from 0.002 µg/g in farmed *Cyprinus carpio* up to 0.011 µg/g in wild *Rutilus frisii kutum* from Iran [40], from 0.025 mg/g dw in muscle tissues of *Oncorhynchus mykiss* up to 0.056 mg/g dw in muscle of *Salmo coruhensis* from Firtina and Güneysu Rivers, Turkey [41], and between 0.18 mg/kg ww in chub (*Leuciscus cephalus*) and 0.36 mg/kg ww in roach (*Rutilus rutilus*) from the Nitra River, Slovakia [42]. Cadmium levels in the present study were in good agreement with reported literature data and with the data from the international organizations.

Chromium is an essential element. But in its hexavalent form it is toxic when ingested. The International Agency for Research on Cancer (IARC) has determined that chromium (VI) compounds are carcinogenic to humans [37]. The minimum level of Cr measured in this study was 0.03 mg/kg ww for the samples of *C. carpio* (Lake Burgas) and *A.brama* (Lake Mandra), whereas the maximum value of 0.06 mg/kg ww was for *C.carassius* (Lake Burgas). In the current study, Cr levels in all samples were lower than the permissible limits set by Bulgarian legislation office (0.3 mg/kg ww) [36]. Cr content in the literature vary between 0.17 mg/kg ww in chub muscle samples and 0.29 mg/kg ww in muscle samples of barbel from Nitra River, Slovakia [42]; between 0.156 mg/g dw and 0.224 mg/g dw in tissues of two trout species from Firtina and Güneysu Rivers, Turkey [43].

Copper is essential for good health, but an excess intake can cause adverse health problems such as liver and kidney damage [44,45]. The copper concentration found in this study was in the range of 0.11 mg/kg ww for *R. rutilus* up to 0.2 mg/kg ww for muscle tissues of *C. carassius*. Relatively low copper concentrations ranging from 0.25 mg/kg ww (perch) to 0.78 mg/kg ww (barbel) were found in samples from Nitra River, Slovakia [42] with significant differences in copper accumulation among the four species (*p* < 0.001). Higher copper concentrations were reported in fish from the Atatürk Dam Lake [46,47] and in fish samples *Prochilodus lineatus, Cyprinus carpio,* and *Mugil cephalus* from the Rio de la Plata River, Argentina (0.44–0.47 μg/g ww) [48]. In this study, the copper content in fish muscle did not exceed the upper limit allowed by Codex Alimentarius and Bulgarin Food Codex (10.0 mg/kg ww) [38].

The mean Fe levels in muscle ranged from 1.68 mg/kg ww for *R. rutilus* to 5.86 mg/kg ww for *A.brama*. Iron is essential for the proper human blood system function since it is found as hemoglobin or myoglobin and as ferritin and hemosiderin in fish. The maximum permitted iron level established by FAO/WHO is 100 mg/g [35]. There is no maximum permitted level for Fe in fish samples according to European legislation, [33] but in the literature the data related with iron concentration are within or higher than the values from the current study [43,49,50,51].

The average Mn levels in the samples were between 0.05 mg/kg ww for *C. carpio* and 0.71 mg/kg ww for *C. carassius*, and both samples were collected from Bourgas Lake. Manganese concentrations muscle tissue of freshwater fishes in the literature has been reported in the range of 9.6–64.3 µg/g for five fish species including *C. carpio* and *C. gibelio* collected from six lakes in Tokat, Turkey [51], 1.450–2.690 for farmed and wild *C. carpio* and *R. frisii kutum* from south-western Caspian Sea areas of Iran [49] and below the detection limit in carp from Beyşehir Lake in Turkey [52]. There are no data about the maximum Mn level permitted according to Bulgarian Food Codex [36]. The data from current study are within those found in the literature.

Ni is a serious pollutant in aquatic environments. Its toxicity leads to cancerous respiratory effects, and affects the immune and reproductive system [37]. The minimum concentration was found in *A. brama* (0.06 mg/kg ww), whereas the maximum was found in *R. rutilus* (0.11 mg/kg ww). Nickel concentrations fluctuated between 0.07 mg/kg ww (chub) and 0.25 mg/kg ww (barbel) for the sample from Nitra River, Slovakia [42], around 0.2 µg/g for *C. carpio* in the Zabol Chahnimeh Reservoirs, Iran [53]. The maximum Ni level permitted for muscle meat of some marine fish is 0.5 mg/ kg ww according to the Bulgarian Food Regulation [36] and 0.4 mg/ kg dw according to European legislation [33]. The concentrations of Ni in our study were below that reported in the literature and various health organizations.

The maximum and minimum level of Pb was 0.27 mg/kg ww for *C. gibelio* and 0.20 mg/kg ww for *C. carpio*, respectively. According to European Commission Regulation (1881/2006/EC) [35], the maximum acceptable concentration (MAC) for Pb in fish meat is 0.3 mg/kg ww. National regulation of the Republic of Bulgaria also prescribed a value of 0.3 mg/kg ww in fresh fish meat [36]. In the literature, the mean concentration of Pb ranged from 1.37 mg/kg up to 12.87 mg/kg in six fish species (*B. grypus*, *B. luteus*, *B. sharpeyi, C. carpio, L. abu* and *S. trisostegus*) in the most important and largest wetland in south-western Iran [40]; 0.31–1.8 mg/kg ww for *L. cephalus*, *B. barbus*, *R. rutilus* and *P. fluviatilis* from the Slovak Nitra River [42]. Lead concentration was below detection thresholds in samples of pontic shad (*A. immaculata*) caught from the Danube River waters [39], in samples of two trout species from the Firtina and Güneysu Rivers in Turkey [43] and in *Acanthobrama marmid, Chalcalburnus mossulensis, Chondrostoma regium, Carasobarbus luteus, Capoetta trutta* and *Cyprinus carpio* from the Atatürk Dam Lake, Turkey [46]. The results from the current study are within the MAC and the data in the literature.

Zn concentration in the current analysis ranged between 1.94 mg/kg ww for *A. brama* and 9.06 mg/kg ww for *C. carassius*. Papagiannis et al. [40] found higher mean zinc concentrations in *R. ylikiensis* than in *C. carpio* and *C. gibelio* and the lowest concentration detected in *S. aristotelis* from Lake Pamvotis (Greece), between 23.37 and 45.51 mg/kg in six freshwater fish species from Iran [38] and between 18.07–21.94 mg/g dw in tissues of two trout species from Firtina and Güneysu Rivers in Turkey [43]. According to the European Commission Regulation (1881/2006/EC) [33], there is no value set for MAC for Zn in fish meat, but Bulgarian Food Codex [36] prescribed a value of 50 mg/kg ww. Limits recommended by the Food and Agriculture Organization for Zn is 30 mg/kg ww [41]. These results are in good agreement with the data within literature and those set by various health organizations.

### 3.2. Potential Health Risk Assessment

The target hazard quotients for As, Cd, Cr, Cu, Fe, Mn, Ni, Pb and Zn, hazard index and target risk for As, Pb and Ni, separately for both target groups (females and males), estimated through the consumption of the five freshwater fish species are shown in Table 5. In this study the THQ and HI for both males and females were less than 1 for all elements. Therefore, there is no non-carcinogenic health risk from ingestion of these metals individually and collectively through the consumption of those five freshwater fish species. Males exhibit a significantly higher HI value than females. Since the arsenic concentration is under the limit of detection for this element, the THQ and TR calculations were not performed. The highest THQ was in *C. gibelio* (0.052 for females and 0.043 for males for Fe which is lower than the acceptable limits). Barone et al. [30] found in their study that the THQ_Hg_ and THQ_Cd_ were lower than 1 for all marine species tested (fish: 0.04–0.80; cephalopods: 0.03–0.09; crustaceans: 0.02–0.05). THQ_Cd_ varies between 0.14–0.16, THQ_Cu_ = 0.01, THQ_Pb_ = 0.001, THQ_cr_ between 0.23–0.34, THQ_As_ between 0.07–0.18, THQ_Ni_ between 0.001–0.002 and THQ_Zn_ between 0.010–0.011 for Nile tilapia (*Oreochromis niloticus*), African sharp tooth catfish (*Clarias gariepinus*), and African big barb (*Barbus intermedius*) caught from Lake Hawassa, Ethiopia [54].

TR was performed for As, Ni and Pb since only those elements are classified as cancerogenic. The values of TR must be equal or lower than 10^−6^ for carcinogens and may be up to 10^−4^. The calculated values in this study are within this range, suggesting that the intake of As, Ni and Pb by consumption of these freshwater fish species would not result in appreciable hazard risk on the human body.

## 4. Conclusions

Some toxic and essential element concentrations were determined in five freshwater fish species collected from Lake Mandra and Lake Bourgas (Bulgaria). The data presented show that these levels are within the maximum acceptable limits set by various health organizations.

No toxic and essential elements were found to be considered as potential health hazards for consumers based on the results from the study. The calculations from THQ and HI showed that these pollutants pose no potential health risk through the absorbed pathway, which means that adverse effects will not occur. The target risk value for toxic inorganic As, Pb and Ni was below 10^–6^, indicating no carcinogenic risk. According to these results, the consumption of these five freshwater fish species is safe for human health.

## Figures and Tables

**Table 1:** Biometrics data (mean ± SD) of freshwater fishes from the Black Sea lakes.

Sample	Location	N	Weight (kg) ± SD	Length (cm) ± SD
Crucian carp (*Carassius carassius*)	S1	6	0.253 ± 0.112	15 ± 2
Common Carp (*Cyprinus carpio*)	S1	3	2.189 ± 0.859	46 ± 9
Roach (*Rutilus rutilus*)	S2	3	0.963 ±0.026	25 ± 9
Freshwater Bream (*Abramis brama*)	S2	4	2.562 ± 1.002	50 ± 5
Prussian Carp (*Carassius gibelio*)	S2	3	1.106 ± 0.089	38 ± 3

S1 (Lake Burgas), S2 (Lake Mandra).

**Table 2:** Microwave digestion system general and operational parameter.

Microwave Digestion System “MARS 6”, Acid Mixture		Step	Initial Power (W)	Time (min)	Final Power (W)	Fan
T °C (max)	200 °C	1	100	5	600	1
Pressure (max)	75 bar	2	600	5	600	1
Quartz vessels	EasyPrep^TM^Plus	3	600	5	800	1
Sample amount	1 g	4	800	15	800	1

**Table 3:** Instrumental parameters for ICP-OES.

Plasma gas flow	8 L/min
Auxiliary gas flow	0.4 L/min
Nebulizer gas flow	0.6 L/min
RF Power	1500 watts
Viewing height	15 mm
Purge flow	High
Plasma view	Axial
Read delay	90 s
Read parameters	Auto (1 to 5 (Min-Max))
Peristaltic pump flow rate	1.5 mL/min
Processing peak	Area/High
Calibration	Linear Calculated Intercept
Spray chamber	Cyclonic Glass
Nebulizer	Concentric Glass, MEINHARD^®^ Type C
İnjector	Alumina 2.0 mm i.d
Quartz torch	1-slot
Auto integration(min-max)	0.1–0.5 s
Replicates	3
Background correction	1 or 2 points, manually

**Table 4:** Concentration of heavy metals (mean ± standard deviation in mg/kg ww) in freshwater fish species from Lake Mandra and Lake Bourgas (Black Sea, Bulgaria).

Toxic and Essential Elements	S1			S2		National and International Standard Limits *
	*C. carassius*	*C. carpio*	*R. rutilus*	*C. gibelio*	*A. brama*	
As	nd	nd	nd	nd	nd	2.0 [32]
Cd	0.04 ± 0.01	0.03 ± 0.001	0.05 ± 0.01	0.04 ± 0.01	0.02 ± 0.01	0.05 [33]
Cr	0.06 ± 0.01	0.03 ± 0.01	0.05 ± 0.01	0.04 ± 0.01	0.03 ± 0.01	-
Cu	0.20 ± 0.02	0.16 ± 0.02	0.11 ± 0.03	0.17 ± 0.11	0.12 ± 0.11	1.0 [34]
Fe	1.85 ± 0.71	1.92 ± 0.15	1.68 ± 0.96	2.23 ± 1.63	5.86 ± 1.97	100 [35]
Mn	0.71 ± 0.02	0.05 ± 0.01	0.19 ± 0.16	0.20 ± 0.10	0.32 ± 0.03	-
Ni	0.08 ± 0.01	0.08 ± 0.01	0.11 ± 0.03	0.09 ± 0.05	0.06 ± 0.01	0.5 [36]
Pb	0.21 ± 0.01	0.20 ± 0.02	0.25 ± 0.06	0.27 ± 0.07	0.15 ± 0.02	0.3 [33]
Zn	9.06 ± 0.44	3.27 ± 0.06	4.99 ± 4.12	6.98 ± 0.66	1.94 ± 0.16	100 [34]

Results represent mean values ± standard deviation (n = 3); nd—not detected; values in a row not sharing a common superscript are significantly different (*p* < 0.05); S1 (Lake Burgas), S2 (Lake Mandra). * The standard limits are for marine fish.

**Table 5:** Risk values (THQ, HI and TR) of each metal contaminant in muscle of five freshwater fish species from lakes near the Black Sea (Bulgaria).

Fish Sample	Target Hazard Quotient (THQ)									Hazard Index (HI)	Target Risk (TR)		
	As	Cd	Cr	Cu	Fe	Mn	Ni	Pb	Zn		As	Pb	Ni
FEMALES													
*C. carassius*	---	0.008	0.004	0.0011	0.043	0.0010	0.0008	0.011	0.006	0.075	---	1.84 × 10^−5^	3.13 × 10^−5^
*C. carpio*	---	0.006	0.002	0.0008	0.044	0.0001	0.0008	0.010	0.002	0.067	---	1.91 × 10^−5^	3.25 × 10^−5^
*R. rutilus*	---	0.010	0.003	0.0006	0.039	0.0003	0.0011	0.013	0.004	0.071	---	6.00 × 10^-5^	4.35 × 10^−5^
*C. gibelio*	---	0.009	0.003	0.0009	0.052	0.0003	0.0010	0.014	0.005	0.085	---	6.51 × 10^−5^	3.82 × 10^−5^
*A. brama*	---	0.004	0.002	0.0006	0.137	0.0005	0.0006	0.008	0.001	0.155	---	3.55 × 10^−5^	2.25 × 10^−5^
MALES													
*C. carassius*	---	0.006	0.003	0.0009	0.035	0.0009	0.0007	0.0093	0.0052	0.078	---	1.59 × 10^−5^	2.51 × 10^−5^
*C. carpio*	---	0.005	0.002	0.0007	0.036	0.0001	0.0007	0.0086	0.0019	0.099	---	1.76 × 10^−5^	2.61 × 10^−5^
*R. rutilus*	---	0.008	0.003	0.0005	0.032	0.0002	0.0009	0.0109	0.0029	0.148	---	4.81 × 10^−5^	3.49 × 10^−5^
*C. gibelio*	---	0.007	0.003	0.0007	0.043	0.0002	0.0008	0.0118	0.0040	0.096	---	5.22 × 10^−5^	3.06 × 10^−5^
*A. brama*	---	0.003	0.002	0.0005	0.113	0.0004	0.0005	0.0065	0.0011	0.082	---	2.85 × 10^−5^	1.80 × 10^−5^

## Data Availability

Not applicable.
